# Microbiota Modulates the Immunomodulatory Effects of Filifolinone on Atlantic Salmon

**DOI:** 10.3390/microorganisms8091320

**Published:** 2020-08-30

**Authors:** Mick Parra, Daniela Espinoza, Natalia Valdes, Rodrigo Vargas, Alex Gonzalez, Brenda Modak, Mario Tello

**Affiliations:** 1 Laboratorio de Metagenomica Bacteriana, Centro de Biotecnología Acuicola, Universidad de Santiago, Alameda, Estación Central, Santiago 9170002, Chile; mick.parra@usach.cl (M.P.); daniela.espinoza@usach.cl (D.E.); natalia.valdes.p@usach.cl (N.V.); rodrigo.vargas.c@usach.cl (R.V.); 2 Laboratorio de Productos Naturales, Centro de Biotecnología Acuicola, Universidad de Santiago, Alameda, Estación Central, Santiago 9170002, Chile; 3 Departamento de Ciencias Biológicas y Biodiversidad, Laboratorio de Microbiología Ambiental y Extremofilos, Universidad de los Lagos, Osorno 5290000, Chile; alex.gonzalez@ulagos.cl

**Keywords:** microbiota, Atlantic salmon, immunomodulatory, interferon gamma, filifolinone

## Abstract

Filifolinone is an aromatic geranyl derivative, a natural compound isolated from *Heliotropum sclerocarpum*, which has immunomodulatory effects on Atlantic salmon, upregulating cytokines involved in Th1-type responses through a mechanism that remains unknown. In this work, we determined whether the immunomodulatory effects of filifolinone depend on the host microbiotic composition. We evaluated the effect of filifolinone on immune genes and intestinal microbiotic composition of normal fish and fish previously treated with bacitracin/neomycin. Filifolinone induced the early expression of IFN-α1 and TGF-β, followed by the induction of TNF-α, IL-1β, and IFN-γ. A pre-treatment with antibiotics modified this effect, mainly changing the expression of IL-1β and IFN-γ. The evaluation of microbial diversity shows that filifolinone modifies the composition of intestinal microbiota, increasing the abundance of immunostimulating organisms like yeast and firmicutes. We identified 69 operational taxonomic units (OTUs) associated with filifolinone-induced IFN-γ. Our results indicate that filifolinone stimulates the immune system in two ways, one dependent on fish microbiota and the other not. To our knowledge, this is the first report of microbiota-dependent immunostimulation in Salmonids.

## 1. Introduction

Aquaculture is one of the faster growing food industries in the world. Currently, aquaculture produces almost the same amount of fish as does pelagic fishing. According to the Food and Agriculture Organization (FAO), aquaculture will be the main source of protein for humans and domestic animals by 2050 [1,2]. One of the main problems that must be overcome for aquaculture to meet this target is diseases caused by pathogenic outbreaks [3]. In the Chilean salmon farming industry, such outbreaks are mainly prevented by antibiotic vaccines [4].

Vaccinating Salmonids has had varying results, however, it is generally accepted that fish lack strong immune memory, and thus require successive stimulation to confer effective protection against pathogens [5,6]. It is quite different with mammals; whose immunological memory confers protection for several years after vaccination. Comparative immunology suggests the difference lies in the nature of the salmonid immune system, which compared to that of mammals has a stronger primary cellular immune response, and a weaker secondary immune response in terms of the abundance, diversity, and affinity of the antibodies produced against the antigens used with conventional methods of vaccination [7].

A successful vaccination requires that the immune system be adequately stimulated by the vaccine. This is achieved with mammals by co-administering the antigen with an adjuvant that stimulates the cellular component of the humoral response [8]. Vaccinations of fish also involve the use of adjuvants to improve the immune response against the antigen [9], most based on adjuvants previously developed for use with mammals.

In recent years, the development of adjuvants for fish vaccination has focused on identifying new immunostimulatory molecules that, together with antigens, can improve immune response [10]. These molecules are generally derived from pathogens and target pattern recognition receptors (PRRs) in antigen-presenting cells [9].

**Figure 1:**
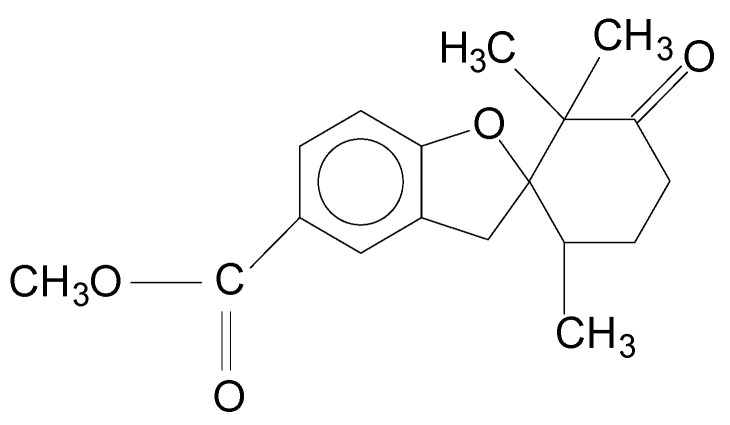
Filifolinone structure.

In recent years, the immunostimulatory potential of several natural products derived from plants has been studied with mammals [11,12] and fish [13], and even used as adjuvants in vaccines [14]. In our laboratory, we have studied the immunostimulatory properties of filifolinone (Figure 1), a natural aromatic geranyl derivative that increases the expression of pro-inflammatory and anti-inflammatory cytokines in SHK-1 cells and up-regulates cytokines involved in Th1-type responses in Atlantic salmon [15,16]. Filifolinone also has adjuvant properties that stimulate the expression of Interferon gamma [17]. The mechanism by which filifolinone acts as an immunostimulant in Atlantic salmon remains unknown.

Studies with mammals have shown that microbiota composition is important in several biological effects on hosts, including immune response [18,19]. Microbiota modulates the metabolism of xenobiotics in mammals, among them orally or intravenously administered drugs and molecules with pharmaceutical properties [20], and influences the efficacy/potency, and toxicity of chemo and immunotherapeutic drugs used to treat cancer [21]. For example, the anti-tumor effect of treatment with CpG oligodeoxynucleotides and antibodies against IL10 is reduced in mice previously treated with antibiotics [22]. This treatment requires the microbiota-dependent production of TNF-α by dendritic cells [22]. Microbiota enhances the efficacy of cyclophosphamide, Ipilimumab (Anti-CTLA4), anti-PD-L1 stimulating the Th1 response [23,24,25]. Cyclophosphamide stimulates the Th17 response in a microbiota-dependent manner, promoting the selective translocation of some Gram-positive bacteria to mesenteric lymph nodes [26]. The interaction between drugs and microbiota seems to be bi-directional, as indicated by changes in the microbial composition associated with the administration of non-antibiotics, and chemo and immune therapeutic drugs [21,23,24].

Based on the above, we determined in this work whether the immunostimulatory properties of filifolinone depend on host microbiotic composition. To do this, we modified the microbiota composition (Dysbiosis), by administering non-absorbable antibiotics at the mucosa level, and then analyzed the action against intestinal microorganisms and changes in the immunomodulatory effect of filifolinone.

## 2. Materials and Methods

### 2.1. Extraction and Isolation of the Natural Compound Filifolinone

Filifolinone was obtained from *Heliotropium sclerocarpum* as described in Parra et al. 2018 [17]. Briefly, *H. sclerocarpum* was collected in Huasco, Chile. The fresh plant was dipped in dichloromethane for 30 s. The organic extract was concentrated to yield a resinous exudate. The resin was separated by chromatographic column. One of the majority fractions was purified by thin-layer chromatography on silica gel, to yield a white solid. Infrared (IR) and proton nuclear magnetic resonance (1HNMR) spectra were obtained. Filifolinone was identified by comparing its spectroscopic data with descriptions in the literature and by co-chromatography with a verified sample.

### 2.2. Fish and Maintenance

One hundred and fifty pre-smolt Atlantic salmon (*Salmo salar*) weighing about 25 g were used. The fish were acclimated for one week before treatment at 12 °C in freshwater aquariums with a biomass of 14 g/L, continuous aeration, and fed with commercial pellets (EWOS MICRO^TM^ 2 mm, Cargill, Coronel, Chile) at 1% of body weight. The fish were maintained in freshwater with a pH between 6.6 and 7, the salinity was adjusted to 6 PSU with NaCl to prevent fungal infection, and total ammonia was maintained in a range below 0.02 mg/L. Seventy percent of the water in all the aquariums was changed every day after feeding. Water parameters were monitored daily prior to and after changing the water. Feeding, changing the water, and measuring water parameters were all done manually. The fish were maintained in accordance with the ethical standards of the Institutional Ethics Committee of the Universidad de Santiago de Chile and the relevant legislation in force. The authorization of the Ethics Committee of the Universidad de Santiago de Chile to perform experiments with fish in the project FONDECYT 1180265 was conceded on 26-01-2018 and is consigned in the report Nº42.

### 2.3. Experimental Design

**Figure 2:**
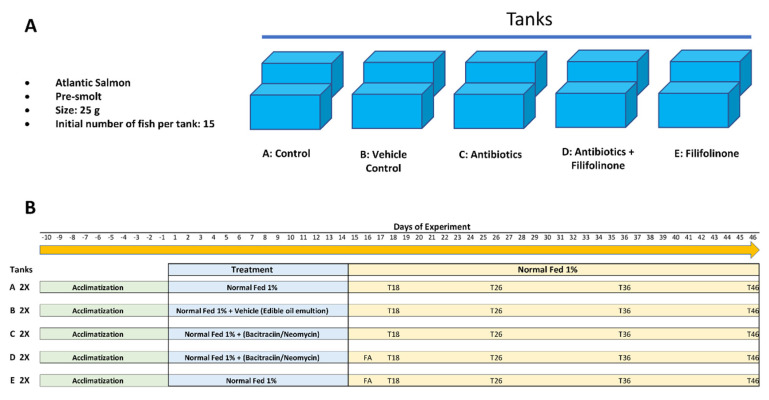
Experimental design. The figure shows the experimental design to test the effects on the immunostimulatory capacity of filifolinone of dysbiosis induced by nonabsorbable antibiotics. (**A**) Shows the conditions to be analyzed, the species, size, and initial number of the fish per tank. Each condition was assessed in duplicate, *n* = 15 per tank and *n* = 30 per treatment; (**B**) Shows the treatments applied per tank and the sampling kinetics. Tanks C and D were treated with nonabsorbable antibiotics for 14 days. On day 16 of experimentation, the fish from tanks D and E (FA) were injected intramuscularly with 100 ug of filifolinone. The sampling days are indicated as T18, T26, T36, and T46.

The fish were divided into five groups, each in duplicate, with 15 fish per tank. Group A: control (untreated). Group B: vehicle control. Group C: fish treated with bacitracin/neomycin. Group D: fish treated first with bacitracin/neomycin, and then with filifolinone. Group D: fish treated only with filifolinone. (Figure 2A)

Group A fish were fed commercial pellets, while Group B fish were fed commercial pellets mixed with edible oil. The fish in Groups C and D were treated daily with 0.4 mg per fish of bacitracin/neomycin (Sigma-Aldrich, St Louis, MO, USA) for 14 days. The antibiotics were emulsified with edible oil to improve their adherence to food pellets. On day 16 of experimentation (the second day after completing the administration of antibiotics), the fish in Groups D and E were injected intramuscularly with 100 μL of L-15 medium containing 1 g/L of filifolinone, thus the total dosage was 100 μg of filifolinone per fish, while fish in groups A, B, and C were injected intramuscularly with 100 μL of L-15 medium to evaluate the effect of the injection on immune parameters. Three fish were sacrificed per aquarium at 14, 18, 26, 36, and 46 days of experimentation (*n* = 6 per treatment), and head kidney and intestine of the fish were removed (Figure 2B). The samples were stored at −80 °C.

### 2.4. Primary Macrophage Culture and Treatment with Filifolinone

Primary macrophage culture was obtained from Atlantic salmon head kidneys, which were removed from fish weighing approximately 50 *g* and disrupted using cell strainer 70 µm in phosphate buffered saline (PBS), supplemented with 2% fetal bovine serum (FBS). The cell suspension was centrifuged at 500× *g* for 10 min at 4 °C. The pellet was resuspended in Leibovitz 15 medium (L15, Gibco, Invitrogen, Carlsbad, CA, USA), supplemented with 10% FBS, (Hyclone, Thermo Fisher Scientific, Logan Utah, UT, USA), 4 mM L-glutamine (Gibco), 50 μM 2-mercaptoethanol (2-ME, Gibco), and 50 μg/mL gentamicin (US Biological, Swampscott, MA, USA). The cells were stained with Tripan Blue and counted using a Neubauer chamber.

Cells (1 × 10^6^) were seeded on 6-well cell culture plates (SPL Life Sciences, Pocheon-si, Korea) and incubated for 4 days in L-15 medium at 16 °C. Subsequently, the cell culture was washed with PBS to remove the erythrocytes in suspension and then the cells were incubated for 2 days in a new L-15 medium. Macrophage-primary culture (MPC) was treated with 5, 10, and 15 μg/mL of filifolinone for 24 h in L-15 medium. To evaluate the effects of the solvent used to solubilize filifolinone, the MPC was also treated with 0.2% DMSO. As positive control of immunostimulation, MPC was transfected with 10 μg/mL Poly I:C (from Polyinosinic-polycytidylic acid, Invitrogen), using X-trem Gen 9 DNA transfection reagent (Roche, Mannheim, Germany), according to the manufacturer’s instructions. As a negative control, a group of cells from MPC remained untreated throughout the experiment. The data are representative of three independent experiments.

### 2.5. RNA Extraction and cDNA Synthesis

To extract RNA, approximately 30 mg of kidney was homogenized in 1 mL of TRIsure (Bioline) and incubated for 5 min at room temperature, then 200 μL of cold chloroform were added and vortexed for 15 s. The samples were incubated for 3 min at room temperature and centrifuged at 12,000× *g* for 15 min at 4 °C. The upper phase was recovered and precipitated with 500 μL of isopropanol, subsequently incubated for 60 min at −20 °C, and centrifuged at 12,000× *g* for 10 min at 4 °C. The supernatant was removed, and the pellet resuspended in 1 mL of ethanol 75% in DEPC water. The samples were centrifuged at 7500× *g* for 5 min at 4 °C. Finally, the pellet was allowed to dry for 10 min and dissolved in 40 μL of DEPC water.

To eliminate contaminating DNA from the samples, 2 μg of RNA were treated with 1 μL of DNAse, RNAse-free (Promega, Madison, WI, USA), and 1 μL of RQ1 DNAse 10× reaction buffer (Promega) in a final volume of 10 μL in DEPC water. The samples were incubated for 30 min at 37 °C, then 1 μL of 25 mM Stop solution EDTA DNAse (Promega) was added and the mixture was incubated again for 10 min at 65 °C. To synthesize cDNA, 0.5 μL of 10 mM dNTPs (IDT DNA), 1 μL of M-MLV (200 μg/μL) (Promega), 1 μL of oligo dT (500 μg/μL) (IDT DNA), 5 μL of M-MLV RT 5× buffer (Promega), and 11 μL of RNA were treated with DNase (Promega) to a final volume of 25 μL in DEPC water. The samples were incubated first at 42 °C for 60 min and then at 70 °C for 15 min, and finally stored at −20 °C.

### 2.6. Analysis of Cytokine Expression by qPCR

Real-time quantitative PCR reactions (qPCR) were performed in 96-well plates (AXIGEN) using the Stratagene Mx 3000P (Agilent technology, Santa Clara, CA, USA). The reaction mixture consisted of 5 μL of SensiMix SYBR^®^ Hi-ROX 2× (Bioline USA, Tauton, MA, USA), 0.5 μM of forward and reverse primers for each analyzed cytokine, 80 ng of cDNA, and ultrapure water (Invitrogen) to complete a final volume to 10 μL. Subsequently, the transcript levels of the cytokines (IL-12, IFN-α1, IFN-γ, IL-1β, TNF-α, and TGF-β) were quantified. For the analyses, the expression of elongation factor 1α (ef1a) was used to normalize the expression of target genes using the ΔΔCT method [27]. Statistically significant differences with respect to the control were determined by a one-way nonparametric *t*-test (Mann–Whitney) (* *p* < 0.05, ** *p* < 0.01, *** *p* < 0.001). The primers used in these experiments are listed in Table 1**.**

### 2.7. DNA Extraction

DNA was extracted from 30 mg of the intestine using the Wizard^®^ Genomic DNA Purification Kit (Promega) following the manufacturer’s instructions. Total DNA was then quantified by UV spectrophotometry using a Tecan INFINITE M200 Pro, standardized at a concentration of 25 ng/μL, and stored at −20 °C

### 2.8. Bacterial Load

The intestinal bacterial load was determined by qPCR using the absolute quantification method. To quantify the 16S copy number, a standard curve was generated from the purified PCR product of a segment between V8 and V9 of the 16S rDNA gene of *E. coli* using the forward primer Bact 1369: 5′-CGGTGAATACGTTCCCGG-3′ and the reverse primer Prok1492: 5′-TACGGCTACCTTGTT-3′ [33]. To quantify the copy number of the 16S gene in each sample, real-time quantitative PCR was performed using SensiFAST™ SYBR^®^ No-ROX Kit (Bioline) in Stratagene Mx 3000p equipment (Agilent Technologies, Santa Clara, CA, USA) and carried out in 96-well reaction plates (PCR^®^ Microplate, Axigen). The reaction mixtures were performed in duplicates using 5 µL of SYBR No-ROX mastermix, 0.5 μM of forward and reverse primer, 25 ng of DNA and ultrapure water (Invitrogen) to complete a final volume of 10 μL. The cycling conditions were: 95 °C for 5 min, followed by 35 cycles for 15 s at 95 °C, 15 s at 60 °C, and 30 s at 72 °C. Data were analyzed using MxPro qPCR software (Agilent Technologies). The statistically significant differences were determined with respect to the control by a one-way nonparametric t-test (Mann–Whitney) (* *p* < 0.05, ** *p* < 0.01, *** *p* < 0.001).

### 2.9. Metagenomic Analysis

The microbial diversity in the intestine was evaluated by amplifying the V3-V4 segment of the 16S rRNA gene using total DNA isolated from this source as a template. The diversity of sequences in this amplicon was assessed by next-generation sequencing using an Illumina platform [34]. The sequence was processed using Qiime 2.0 [35] and DADA2 [36] software following the reported procedure [37,38]. All contigs shorter than 250 base pair (bp) or longer than 465 bp were eliminated. Identical sequences were clustered to reduce the required computer load. Finally, the operational taxonomic units (OTUs) were assigned using the SILVA Database v132 [39,40]. The taxonomic assignment was made according to the percentage of alignment with a minimum of 97% of identity for accurate assignment.

## 3. Results

### 3.1. Effect of Filifolinone on the Immune Status of Atlantic Salmon

**Figure 3:**
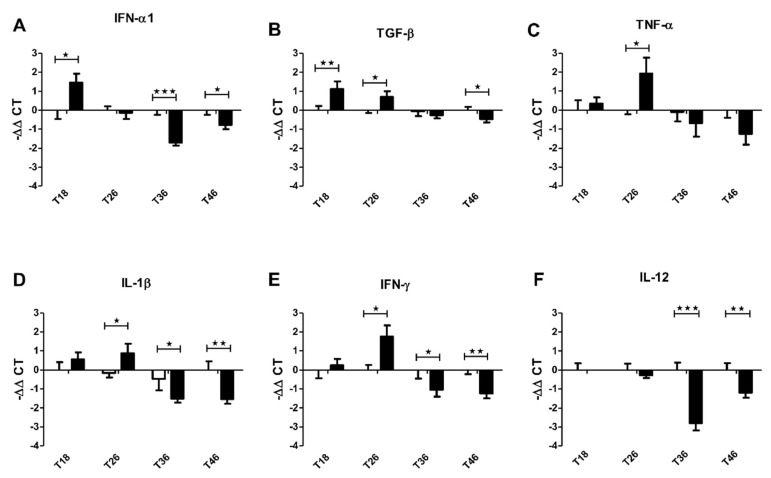
Kinetics of the immunomodulation effect of filifolinone on Atlantic salmon. The figure shows the changes in the expression of pro and anti-inflammatory cytokines at 2, 10, 20, and 30 days post-intramuscular injection of 100 µg filifolinone (T18, 26, 36, and 46 days of experimentation, respectively). The expressions of (**A**) IFN-α1; (**B**) TGF-β (**C**) TNF-α; (**D**) IL-1β; (**E**) IFN-γ; and (**F**) IL-12 were evaluated using head kidney tissue of fish treated with filifolinone (black bars) and untreated fish (white bars). The expression of immune genes was normalized with respect to the expression of elongation factor 1α (ef1a) and the mean value under the control condition. Statistically significant differences were determined with respect to the control by a one-way nonparametric *t*-test (Mann–Whitney) (* *p* < 0.05, ** *p* < 0.01, *** *p* < 0.001) (Number of tanks = 2, *n* per tank = 3, *n* per treatment = 6).

Previous reports showed that filifolinone has in vitro and in vivo immunomodulatory effects on SHK-1 cells and pre-smolt Atlantic salmon [16,17,41,42]. In both experiments, the effects were evaluated within 48 h post-injection in the in vivo experiment or post exposure in the in vitro experiment. In this work, we evaluated the immunomodulatory effect of filifolinone over a longer period of time by taking samples at 2, 10, 20, and 30 days post-injection (days 18 (T18), 26 (T26), 36 (T36), and 46 (T46) of experimentation, respectively). We focused our analysis on the immunological genes that were previously reported as affected by exposure to or administration of filifolinone. We identified three groups of genes, early response genes (ERG) whose expression changes in the first 2 days post administration of filifolinone (T18, IFN-α1, and TGF-β) (Figure 3A,B), the early-late response genes (ELRG), whose expression changes in 10 days post-administration of filifolinone (T26, TNF-α, IL-1β, and IFN-γ) (Figure 3C–E), and late response genes (LRG) whose expression only changes at 20 and 30 days post-administration of filifolinone (T36 and 46 respectively) (IL-12) (Figure 3F). The expression of early and early-late response genes increased in the first 10 days after filifolinone administration (T18 and T26), which tend to repress values below those of the control by 20 to 30 days post-filifolinone administration (T36 and 46 respectively). In the case of IL-12, we observed that filifolinone alone represses expression (by close to tenfold) and this effect is present in the late responses (Figure 3F). The expression of the early response genes IFN-α1 and TGF-β increased around three times after filifolinone was administered (T18) (Figure 3A,B), while the expression of TGF-β continued to increase until 10 days after administering filifolinone (T26) and returned to its normal level at 20 days post-administration (T36) (Figure 3B). The expression of IFN-α1 followed a similar pattern, but decreased sooner and returned to its normal level at 10 days post-administration (T26) and at 20 days post-administration had decreased fourfold (T36) (Figure 3A). The expression patterns of early-late response genes were similar, with the expression of all of them increasing by two to four times by 10 days post-filifolinone administration (T26). However, the expression of these genes decreased by about three times with respect to the control at 20 to 30 days post-filifolinone administration (T36 and 46, respectively) (Figure 3C–E). The level of expression of the late responsive gene IL-12 was similar to that of the control until 20 days post-filifolinone administration (T36), when its expression decreased by around 8 times, and further decreased by four times by 30 days post-filifolinone administration (T46) (Figure 3F). Together, these results suggest that filifolinone induces effects in a cascade, resulting in changes in fish physiology that continue over time.

### 3.2. Effect of Antibiotics on the Immunomodulatory Capacity of Filifolinone

**Figure 4:**
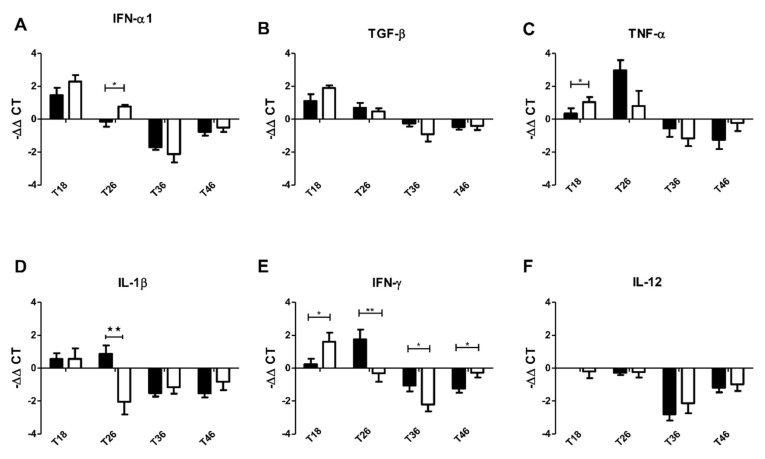
Kinetics of the effect of bacitracin/neomycin on the immunostimulatory capacity of filifolinone in Atlantic salmon. Figure 4 shows the changes in the expression of pro and anti-inflammatory cytokines in fish treated for 14 days with bacitracin/neomycin and subsequently injected intramuscularly with 100 µg filifolinone. The expression (**A**) IFN-α1; (**B**) TGF-β; (**C**) TNF-α; (**D**) IL-1β; (**E**) IFN-γ; and (**F**) IL-12 was evaluated on salmon head-kidney tissue at 18, 26, 36, and 46 days of experimentation in fish treated with bacitracin/neomycin and injected with filifolinone (white bars) and fish only treated with filifolinone (black bars). The expression of immune genes was normalized with respect to the expression of ef1a and the mean value under the control condition. The statistically significant differences were determined with respect to the control by a one-way nonparametric *t*-test (Mann–Whitney) (* *p* < 0.05, ** *p* < 0.01) (Number of tanks = 2, *n* per tank = 3, *n* per treatment = 6).

Some immunotherapeutic drugs that target immune cells stimulate the immune system in a microbiota-dependent way. In order to determine whether immune system stimulation by filifolinone requires elements in Atlantic salmon microbiota, we administered a broad-spectrum mixture of non-absorbable antibiotics, bacitracin/neomycin, for fourteen days. We then administered a dose of filifolinone by intramuscular injection and evaluated the expression of immune genes that respond to filifolinone. We observed that the ELRG (TNF-α, IL-1β, and IFN-γ) and one ERG (IFN-α1), change its patterns of expression (Figure 4A,C–E). We found statistically significant differences in the expression at days T18 (TNF-α), T26 (IL-1β and IFN-γ), and T36 and T46 (IFN-γ).

The mRNA level of the gene encoding for IFN-γ was the most affected of the evaluated genes. Filifolinone induced a peak in expression at T26 in fish without antibiotic treatment and T18 in fish treated with antibiotics and filifolinone. The IFN-γ from fish treated with antibiotics and filifolinone had a level of expression at day T26 similar to of the control fish (without filifolinone treatment), while at T36, IFN-γ expression was almost half that of fish treated with both filifolinone and antibiotic. TNF-α expression had increased almost twofold in fish treated with antibiotics and filifolinone by T18 (2 days post-injection) (Figure 4C). This means that the treatment with antibiotics prior to administering filifolinone changed TNF-α and IFN-γ expression patterns from that of ELRG to that of ERG. IFN-α1 transcript levels were higher by T26 (10 days post-injection) in fish pre-treated with antibiotics followed by filifolinone (Figure 4A) than in fish treated only with filifolinone. IL-1β transcript levels decreased fourfold by T26 in fish previously treated with antibiotics, while the transcript levels of fish injected only with filifolinone increased about threefold (Figure 4D).

### 3.3. The Effect of Non-Absorbable Antibiotics on the Expression of Cytokine in Atlantic Salmon

Antibiotic-dependent changes in the expression patterns of the genes that respond to filifolinone could be the result of a connection between the microbiota and the filifolinone-based stimulation mechanism, although it could also be the direct effect of the antibiotics on the immune system or the immune system response to the dysbiosis induced by the antibiotic mix.

**Figure 5:**
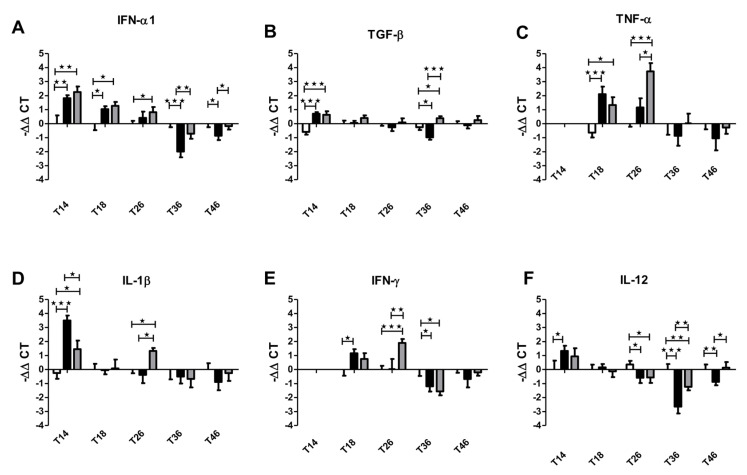
Kinetics of the effect of bacitracin/neomycin on the immune system of Atlantic salmon. The figure shows the changes in the expression of pro- and anti-inflammatory cytokines in fish treated for 14 days with bacitracin/neomycin. The expression of (**A**) IFN-α1; (**B**) TGF-β; (**C**) TNF-α; (**D**) IL-1β; (**E**) IFN-γ; and (**F**) IL-12 in head kidney was evaluated at 14, 18, 26, 36, and 46 days of experimentation of fish treated with bacitracin/neomycin (gray bars), fish treated with vegetable oil (black bars), and untreated fish (white bars). The expression of immune genes was normalized with respect to the expression of ef1a and the mean value under the control condition. The statistically significant differences were determined with respect to the control by a one-way nonparametric *t*-test (Mann–Whitney) (* *p* < 0.05, ** *p* < 0.01, *** *p* < 0.001) (*n* per tank = 3, two tanks, *n* per treatment = 6).

To determine whether antibiotic-dependent changes in the expression patterns of filifolinone-response genes are the result of a connection between the mechanism of filifolinone and microbiotic immunostimulation, we evaluated the effect of antibiotics and their carrier (vegetable oil) on the transcript levels of IFN-α1, TNF-α, IL-1β, IFN-γ, TGF-β, and IL-12. We administered a mixture of non-absorbable antibiotics (bacitracin and neomycin) for fourteen days and analyzed the transcript levels of pro- and anti-inflammatory cytokines at 14, 18, 26, 36, and 46 days of experimentation. We observed that non-absorbable antibiotics increase transcription levels of pro-inflammatory cytokines IFN-α1, TNF-α, IL-1β, and IFN-γ in the first days after antibiotics were administered (T14 to T26) (Figure 5A–D), IFN-α1 transcript levels increased between two and fourfold in fish treated with antibiotics at 14, 18, and 26 days of experimentation. These levels later returned to values similar to those of the control in the last day of experimentation. Interestingly, the transcript levels increased by similar magnitudes on the same days in fish fed with the carrier used in the oral administration of the non-absorbable antibiotics (vegetable oil) (Figure 5A). TNF-α transcript levels in fish treated with antibiotics at 18 and 26 days of experimentation increased four and sixteen-fold, respectively. Although a similar effect was observed in fish treated with oil, with transcript levels increasing at 18 and 26 days of experimentation, these changes also show statistically significant differences from the samples from fish treated with filifolinone (Figure 5C). In the case of IL-1β, transcripts levels increased threefold in fish treated with antibiotics at 14 and 26 days of experimentation. The transcript levels later return to values similar to those of the control. The oil used as a carrier also increased transcript levels around six times, but only after 14 days of experimentation (Figure 5D). A similar effect is observed in transcript levels of IFN-γ, which increased about four times after 26 days of experimentation. Transcript levels subsequently decreased about 3 times at 36 days post-experimentation. The oil used as a carrier also increased transcript levels around 3 times at 18 days of experimentation and decreased around 3 times at 36 days of experimentation (Figure 5E). In the case of IL-12, transcript levels decreased two to four times in fish treated with antibiotics after 26 and 36 days of experimentation, respectively. However, IL-12 transcript levels in fish fed with the carrier also decreased after 26, 36, and 46 days of experimentation (Figure 5F). These results are consistent with our previous results, that showed an inflammatory process associated with the changes in the microbiota composition induced by exposure to antibiotics.

Our results suggest that changes in the pattern of expression of IL-1β and IFN-γ reflect the interaction between microbiota and filifolinone, particularly in expression levels at T26.

### 3.4. Induction of a Filifolinone-Dependent Antiviral Response is Independent of Microbiota

Our results show that filifolinone-induced IFN-α1 is independent of antibiotics. To confirm that filifolinone-dependent IFN-α1 induction does not require a microbial component in Atlantic salmon microbiota, we evaluated the effect of filifolinone on the expression of genes related to the activation of IFN-α1 in primary culture of Atlantic salmon head kidney cells. To this end, cell cultures were treated with 5, 10, and 15 μg/mL of the filifolinone, using cells without treatment and cells treated with DMSO as a control. Poly I: C was used as a positive control, which activates the antiviral immune response by binding to RLR receptors. At 24 h post-treatment, the transcript levels of the receptors responsible for viral recognition, PAMPs RIG-1 and MDA-5, and TLRs like TLR3 and TLR9, were analyzed using RT-qPCR. The transcript levels of the transcription factor IRF3, IFN-α1, and Mx were also determined.

**Figure 6:**
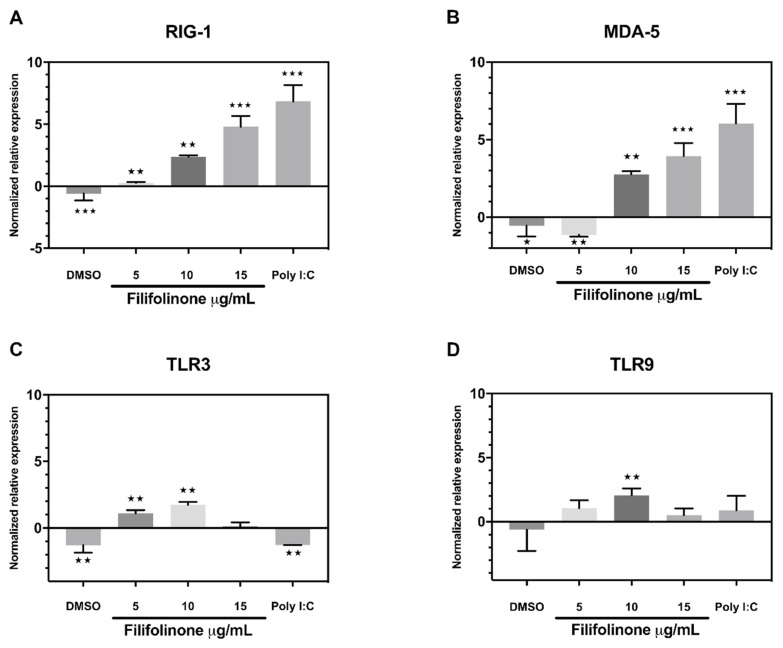
Transcript levels in the gene expression of pattern recognition receptors (PRRs) of innate antiviral immune response in primary head kidney culture from filifolinone-treated Atlantic salmon. The cells were incubated with 5, 10, and 15 µg/mL of filifolinone for 24 h at 16 °C. The graphs show the transcripts levels at relatively normalized expression of (**A**) RIG-1; (**B**) MDA-5; (**C**) TLR3; and (**D**) TLR9 genes evaluated by qPCR. The relative quantification of transcript levels was calculated with the Pfaffl formula. Normalization was carried out in relation to untreated cultures and the constitutive expression gene ef1a (elongation factor 1α). Data are representative of three independent experiments. The statistical differences between untreated and experimental cultures were determined by the Mann–Whitney U test (* *p* < 0.05, ** *p* < 0.01, *** *p* < 0.001).

The transcripts levels of the RIG-1 and MDA5 receptors increased after exposure to filifolinone in a dose-dependent pattern similar to the effect of Poly I: C. The RIG-1 and MDA5 receptors have been identified in many fish species [43]. These receptors are located in the cytoplasm and can be induced in vivo and in vitro by viral pathogens, as well as synthetic dsRNA, poly (I:C), leading to the production of type I interferon (IFN) and the expression of genes stimulated by IFN (ISGs) [44]. The expression of the TLR3 and TLR9 receptors also increase in primary culture after exposure to filifolinone (10 μg/mL) (Figure 6). These receptors, which belong to the TLR family, are highly conserved in the species [45], and recognize viral nucleic acids. Unlike the RLR receptors, the TLR3 and TLR9 receptors are membrane proteins located in endosomes that recognize double-stranded viral RNA [46] and viral DNA [47].

**Figure 7:**
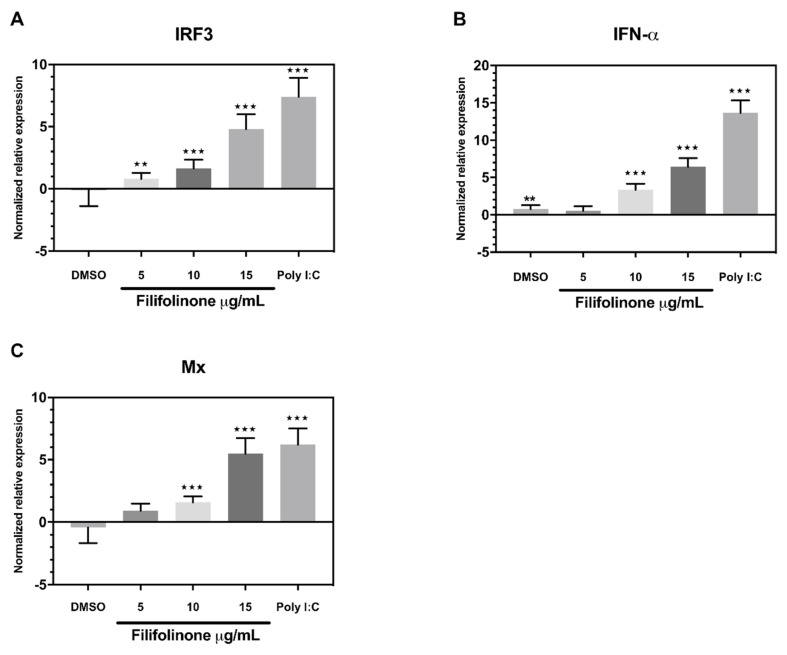
Transcript levels of innate immune response genes in primary culture of Atlantic salmon head kidney treated with filifolinone. The cells were incubated with 5, 10, and 15 µg/mL of filifolinone for 24 h at 16 °C. The graphs show the transcript levels of transcription factor (**A**) IRF3; (**B**) cytokine IFN-α1; and (**C**) protein Mx. The relative quantification of transcript levels was calculated using the Pfaffl formula. Normalization was carried out in relation to the untreated cultures and the constitutive expression gene ef1a (elongation factor 1α). Data are representative of three independent experiments. The statistical differences were determined between untreated and experimental cultures by the Mann–Whitney U test (** *p* < 0.01, *** *p* < 0.001).

Consistent with the observations above, IFN-α1, Mx, and IRF3 also increase in a dose-dependent manner after exposure to filifolinone (Figure 7), which suggests that the induction of IFN-α1 expression and its pathway after filifolinone is administered is independent of a microbial component.

### 3.5. Effects of Filifolinone on Microbiotic Composition

**Figure 8:**
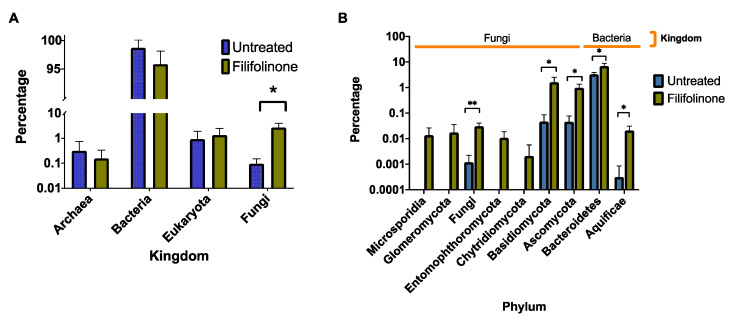
Effect of filifolinone on the composition of the Atlantic salmon gut microbiota. The figure shows the effect of intramuscular injection of filifolinone on the microbiotic composition in the gut epithelia of Atlantic salmon. Microbiota was evaluated by 16S rRNA sequencing of the V3–V4 region using samples of total DNA from gut epithelia. The figure shows (**A**) Kingdoms that had statistically significant changes in abundance; and (**B**) Phyla belonging to the Kingdoms Bacteria and Fungi with statistically significant differences in abundance. Statistical significance was determined by a *t*-test (* *p* < 0.05, ** *p* < 0.01).

Our results show that some immunomodulatory properties of filifolinone require a microbial component in the fish gastrointestinal tract. The participation of a microbial component implies the presence of a microorganism or consortium that in response to filifolinone changes in abundance or secretes compounds/enzymes that interact with the host or filifolinone. To explore this possibility, we analyzed the microbial composition in the gut epithelium of fish treated with filifolinone, control fish without any treatment and fish pre-treated with antibiotics (bacitracin/neomycin) and further stimulated with filifolinone. Samples were taken on day T26 because the participation of microbiota was most clearly observable at that point. We analyzed the effect on microbiome composition by sequencing the 16S rRNA with universal primers, which can also amplify the ribosomal RNA of some eukaryotic microorganisms. Our results show that treatment with filifolinone significantly alters the abundance of fungi (Figure 8A). The total frequency of bacteria did not change significantly in fish treated with filifolinone, although members of the Kingdom Bacteria accounted for more than 95% of abundance. Twenty-two bacterial phyla were identified in samples from fish treated with filifolinone (Proteobacteria, Firmicutes, Actinobacteria, Chlorobia, Cyanobacteria, Bactertoidetes, Verrucomicrobia, Chloroflexi, Plantomycetes, Tenericutes, Acidobacteria, Spirochaetes, Clamydiales, Lentisphaerae, Fusobacteria, Deinococcus_thermus, Chloacimonetes, Nitrospirae, Candidatus Saccharibacteria, Fibrobacteres, Aquificae, and Gemmatimonadetes). The main phyla identified were Proteobacteria (25.28%), Firmicutes (25.37%), and Actinobacteria (16.55%) (Table S1). We found statistically significant differences between fish treated with filifolinone and untreated fish in the abundance of Bacteroidetes and Aquificae, the largest difference being among Bacteroidetes, the abundance of which was approximately twofold in in samples from filifolinone-treated fish (Figure 8B).

**Figure 9:**
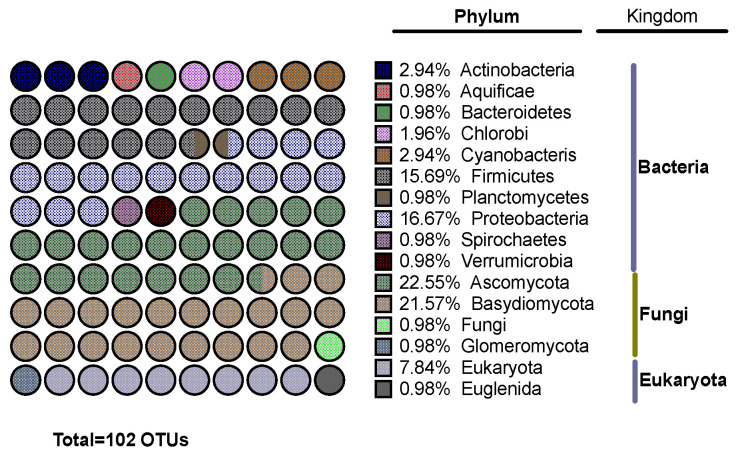
Effect of filifolinone on OTU abundance. The figure shows the distribution among kingdom and phyla of OTUs with statistically significant differences in the abundance between filifolinone-treated fish and untreated fish. A total of 102 OTUs showed differences in abundance (*p* < 0.05), most of these being from the Kingdoms Bacteria and Fungi. Statistical significance was determined by a *t*-test with *p* < 0.05.

We identified a total of 1752 OTUs in filifolinone-treated fish and 1687 in untreated fish. With filifolinone treated-fish, 449 OTUs represented 95% of total abundance, while there were only 167 OTUs among untreated fish (Figure S1). This suggests that filifolinone increases microbial diversity. An analysis of OTUs associated with filifolinone administration (*p* < 0.05) revealed 102 OTUs, belonging mainly to the Kingdoms Bacteria (45.1%) and Fungi (46.1%). The main bacterial phyla associated with filifolinone administration were firmicutes (15.69%), and proteobacteria (16.67%), while the main fungal phyla were Ascomycota (22.5%) and Basidiomycota (21.57%) (Figure 9) (Table S2). This suggests that although administered to fish by intramuscular injection, filifolinone modifies the composition of intestinal microbiome by a still unknown mechanism that could involve direct interaction with bacteria from fish microbiota or indirectly via modification of the immune system, or both.

### 3.6. Effects of Antibiotics on the Microbial Composition of Filifolinone-Treated Fish

**Figure 10:**
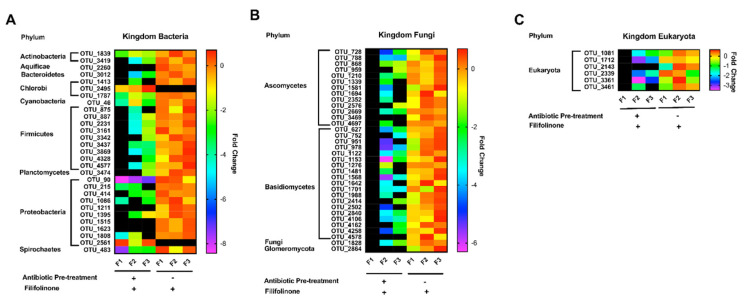
Effect of antibiotics on OTUs that respond to filifolinone. The figure shows the groups of OTUs that respond to filifolinone (102), the abundance of which is affected significantly (*p* < 0.05) by the pre-administration of bacitracin and neomycin (69 OTUs). The figure shows (**A**) OTUs belonging to the Kingdom Bacteria; (**B**) OTUs belonging to the Kingdom Fungi; and (**C**) OTUs belonging to the Kingdom Eukaryote. The abundance of each OTU was normalized by the highest mean, either from samples from fish treated with filifolinone or samples from fish pre-treated with antibiotics before filifolinone was administered. The relative abundance is shown on a color scale. Statistical significance was determined by a *t*-test with *p* < 0.05. Black bars indicate that no OTUs were detected.

Our results suggest that filifolinone requires a microbial component to properly stimulate the immune system. At T26, we identified changed in microbial composition of the gastrointestinal tract from fish treated with filifolinone. To determine if any of these microorganisms are also sensitive to the action of antibiotics, we used 16S rRNA sequencing to determine the microbiota composition of intestinal epithelium of fish treated with antibiotics prior to the administration of filifolinone. Our results showed that 69 of the 102 OTUs (67.6%) that respond to filifolinone have statistically significant differences in abundance in fish treated with both antibiotics and filifolinone. The abundance of most the OTUs (100) being lower in in the latter condition. Twenty-nine OTUs belong to the Kingdom Bacteria, distributed among 9 phyla (Actinobacteria, Aquificae, Bacteroidetes, Chlorobi, Cyanobacteria, Firmicutes, Proteobacteria Planctomycetes, and Spitochaetes) (Figure 10A). The rest of the OTUs belong to Kingdom Fungi (34 OTUs) and Eukaryota (6 OTUs) (Figure 10B,C). Interestingly, some OTUs from the Kingdom Fungi were not detected in fish treated with both filifolinone and antibiotics, suggesting that antibiotics preclude a condition that prevents the growth of these fungi at detectable levels (Figure 10B) (Table S2).

The analysis of the effects of filifolinone and antibiotics on the bacterial loads of fish treated with these compounds did not reveal any detectable change in the bacterial load with either treatment (data not shown), suggesting that some effect on the host is mediated by changes in microbiota composition more than by the absolute abundance of microorganisms.

The 69 OTUs identified as potential targets to isolate allochthonous probiotics to improve the immunomodulatory effect of filifolinone.

### 3.7. Effects of Filifolinone on the Class Clostridia

**Figure 11:**
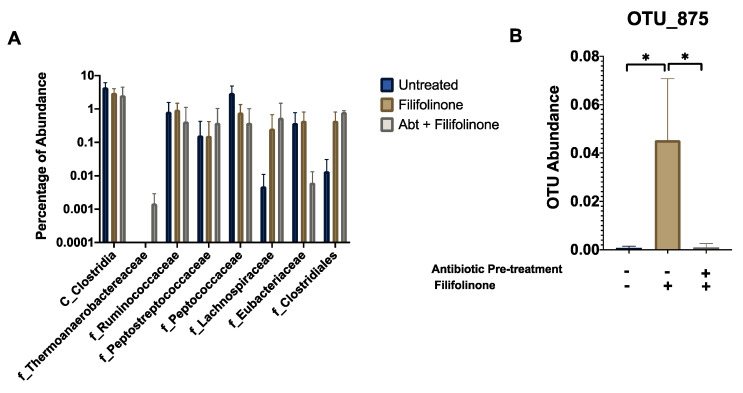
Effect of filifolinone on Class Clostridia. The figure shows (**A**) the relative abundance of the families from the Class Clostridia associated with the production of butyrate in fish pre-treated with antibiotics and stimulated with filifolinone (Abt+ filifolinone), treated with filifolinone, and without treatment (untreated); and (**B**) the abundance (percentage) of OTU_875 (Coprothermobacter spp.) in fish treated with filifolinone, pre-treated with antibiotics and controls (untreated). Statistical significance was determined by a *t*-test with *p* < 0.05 (*).

Antibiotics could prevent the immunomodulatory effects of filifolinone by inducing an increase in bacteria or consortium that produce immunosuppressive molecules. Although the nature of the chemical crosstalk between microbiota and host is not totally understood, butyrate has been identified as a key microbial molecule with pleiotropic effects that induce an anti-inflammatory stage in the gastrointestinal tract, allowing the proper interaction between microbiota and the immune system [48]. Butyrate is produced mainly by bacteria from the class Clostridia. Thus, an increase in this group implies a better capacity to produce butyrate. We evaluated the abundance of OTUs belonging to families of this class associated with butyrate production [49] among control, and filifolinone-treated fish and found that under control conditions, Clostridia represent around 4.4% of detected OTU abundance, while in fish treated with filifolinone, Clostridia represent 3.0%. Abundance decreases close to 2.6% in fish treated with antibiotics and filifolinone. While these results represent a tendency, the differences were not statistically significant (Figure 11A). Among the OTUs with statistically significant differences between control, filifolinone, and filifolinone-antibiotic treatments was the OTU from the class Clostridia, OTU875, which was less abundant in fish treated with antibiotics and filifolinone (Figure 11B) (Table S2). The importance of this OTU in the production of butyrate and in the effects of filifolinone on Atlantic salmon remains to be determined.

## 4. Discussion

Pharmacomicrobiomics is an emerging field that explores the role of microbiota in the effectiveness of drug toxicity [50]. Most of the current knowledge about this interaction comes from studies with chemo and immunotherapeutic drugs in mammals [21]. This amazing property relies on the huge metabolic diversity of microbiota [51] and the complex interaction between microbiota and the immune system [52]. This interaction not only affects orally administered drugs, like metformin, which selective modification of microbiota is necessary to explain its antidiabetic properties [53], but also affect drugs administered intravenously like chemo and immunotherapeutic. Irinotecan and methotrexate are examples of toxic drugs modified by microbiota. Irinotecan is inactivated in the liver by glucuronidation and eliminated by biliary excretion. This metabolite is reactivated by β-glucunoridase activity produced by microbiota, which affects the viability of gut epithelial cells [54,55]. Microbiota modulate the toxicity of methotrexate through by stimulating TLR2 in epithelial cells, which induce the expression of a multidrug resistance pump and consequently improve the efflux from epithelial cells [56]. The mechanisms of interaction of immunotherapeutic drugs with microbiota is more complicated, because with some drugs this mechanism involves increasing the permeability of the intestinal barrier with the translocation of Gram-positive bacteria to lymph nodes and an increase in the expression of IFN-γ and polarization toward Th1 lymphocytes [23]. With other drugs, the mechanism involves microbiota-dependent stimulation of TNF-α secretion by dendritic cells [22].

Our results indicate that the immunomodulatory properties of filifolinone are in two forms. The first is the stimulation of antiviral response mediated by the increase in the expression of PRR and its downstream pathway. Although it is unknown if filifolinone binds to these receptors associated with antiviral response, in silico studies have shown the affinity of filifolinone for TLR1-2 heterodimer, both involved in the recognition of lipopeptides [57]. Further studies are required to evaluate filifolinone’s affinity for the PRR associated with antiviral response. The increase in IFN-α1 in fish treated with antibiotics and filifolinone, and its stimulation in primary cultures in an environment free of microbial components suggest that stimulation of antiviral response by filifolinone is independent of microbiota composition.

Studies indicate that filifolinone stimulates the immune system by polarizing Th1 lymphocytes [16]. Our results suggest that this second form of immunostimulation is mediated by microbiota. In particular, a filifolinone-dependent increase in IFN-γ is prevented by prior administration of antibiotics. A microbiota-dependent increase in IFN-γ expression has been observed in cyclophosphamide [24], anti-CTLA4 [23], and anti-PD1-L1 [25,58,59,60]. The increase with cyclophosphamide is due to the translocation to lymph nodes of Gram-positive bacteria, stimulating a polarized Th1 response [24,61]. This has not been reported with Anti-CTLA4 or Anti-PD1-L1, but it is plausible that this occurs with both due to inflammation of the gut, infiltration of lymphocytes and loss of mucosa, which results in severe colitis during therapy [62]. The role of filifolinone in inducing the translocation of bacteria to head-kidney or the spleen has not been studied, but it is interesting that the capacity of filifolinone to stimulate IFN-γ expression has only been observed in experiments using fish and not in assays with cell cultures [15,16,17,42], which suggests that an additional in vivo factor is necessary to increase IFN-γ expression.

Microbiotic diversity increased as a result of exposure to filifolinone, which was also observed after chemo and immunotherapeutic drugs were administered intravenously [21]. In mammals, intravenous drugs are inactivated in the liver and secreted together with bile acids to the gastrointestinal tract where they interact with the microbiota and gut cells [20]. If a similar mechanism exists in fish, it can be supposed that intramuscular filifolinone can modify microbiota by being excreted to the gastrointestinal tract. Although there is no report that directly addresses the role of microbiota in the Atlantic salmon immune system, an experiment with gnotobiotic zebrafish showed that microbiota is necessary to induce the expression of more than 200 genes related to innate immunity [63,64]. Moreover, experiments using probiotics have shown that yeast and lactic acid bacteria can improve the function of the Atlantic salmon immune system [65,66,67] and other fish [68]. Filifolinone also has antibacterial activity against *Staphylococcus aureus* and *Salmonella thiphymurium* [69]. Thus, some changes in microbiota could be the consequence of this activity against Gram- positive or negative bacteria in the Atlantic salmon gastrointestinal tract.

We found that intramuscular injection of filifolinone increases the abundance of microorganisms belonging to the Kingdom Fungi and phyla Bacteroidetes. Fungi (yeast) are rich in β-glucans, which are recognized stimulators of the immune system in fish [66,67,70]. The genus Bacteroides, which belongs to the phylum Bacteroidetes, has been associated with the effectiveness of anti-CTLA-4 in mammals by stimulatingTh1 activation and maturation of dendritic cells [23]. To identify the group of microorganisms responsible for this stimulation, we modified the microbiota using a broad spectrum of antibiotics. This strategy has been used with mammals and fish to identify biological effects related to microbiota, including drugs and probiotics used as immunostimulants [71,72,73]. Our analyses indicate an association between the effect of filifolinone on microbiota and immune stimulation and 69 OTUs, most of which are bacterial or fungal. Among the interesting phyla associated with filifolinone-dependent induction of IFN-γ are firmicutes. Some members of this phylum belong to classes Bacilli and Clostridia, which have immunomodulatory properties. We identified an association with OTUs assigned to both classes. Clostridia is an interesting group because the abundance of these bacteria increases in mammals as a consequence of treatment with anti-CTLA-4 or anti-PD1 [74,75]. Some members of the class Clostridia produce butyrate, a post-biotic with strong anti-inflammatory activity that promotes Treg differentiation [76]. Although it is not well understood how Clostridia improves the efficacy of anti-CTLA-4 or anti-PD1, it is hypothesized that clostridia increases stimulation of TIL mediated by effector T-Cell [23,75]. Low concentrations of butyrate only stimulate Treg differentiation in the presence of TGF-β. In the absence of this cytokine, and a high concentration of butyrate, Treg differentiate to a Th1- like IFN-γ-producing Treg [77,78,79]. This condition is not consistent with what is observed in T26, where IFN-γ expression is associated with a slight increase in the of transcription level of TGF-β, with no observed changes in the abundance of butyrate-producing bacteria. Future characterization of butyrate concentration in serum and T-cell differentiation in Atlantic salmon based on the development of specific antibodies such in mammals, will help to clarify the mechanism by which microbiota increase IFN-γ expression.

This work shows a connection between microbiota and the immunostimulant properties of filifolinone, but the precise mechanism of interaction remains to be determined. The isolation of bacteria from Atlantic salmon microbiota that can improve the effect of filifolinone could open a new line of study to increase the potency of vaccines used in salmon farming. Further studies are required to probe this possibility.

## 5. Conclusions

Our results indicate that the immunomodulatory properties of filifolinone involve two forms of stimulation. One direct, likely based on the interaction with a receptor, that explains the increase of IFN-α1 and TNF-α, and a secondary microbiota-dependent response based on expression of IFN-γ. Further studies are required to determine this mechanism and identify some autochthonous probiotic bacteria that can improve the properties of filifolinone. To our knowledge, this is the first report in Salmonids of a physiological effect that is dependent on microbiota composition, suggesting that microbiota is a key element to take into account in the design of effective immunostimulatory treatments to efficiently fight against pathogens that infect Atlantic salmon and other Salmonids.

## Figures and Tables

**Table 1:** Primers used to evaluate the expression of the immune response by RT-qPCR.

Gene	Sequence	Ref
ef1a	F: 5′-GGGTGAGTTTGAGGCTGGTA-3′ R: 5′-TTCTGGATCTCCTCAAACCG-3′	[28]
IL-12	F: 5′-AATCAGCTGTCGAGCCAA-3′ R: 5′-GAAGGGACCAGGGGGTCT-3′	Imarai M. Laboratory
IFN-γ	F: 5′-CCGTACACCGATTGAGGACT-3′ R: 5′-GCGGCATTACTCCATCCTAA-3′	[28]
TGF-β	F: 5′-AGCTCTCGGAAGAAACGACA-3′ R: 5′-AGTAGCCAGTGGGTTCATGG-3′	[28]
IL-1β	F: 5′-CCCCATTGAGACTAAAGCCA-3′ R: 5′-GCAACCTCCTCTAGGTGCAG-3′	[28]
TNF-α	F: 5′-AGGCTTTTTCCCAGGGC-3′ R: 5′-GACTCCGAATAGCGCCAA-3′	[16]
IFN-α1	F: 5′-GGACAAGAAAAACCTGGACG-3′ R: 5′-CGTTGATGTCAAACGGTTTCT-3′	[28]
MX	F: 5′-TGTAACACGATGCCCTCTCG-3′ R: 5′-GACGTCAGGGGAGCCAATC-3′	Imarai M. Laboratory
IRF3	F: 5′-TGGACCAATCAGGAGCGAAC-3′ R: 5′-AGCCCACGCCTTGAAAATAA-3′	[29]
RIG-1	F: 5′-GTCAGCAGCCCAGGTGTTTCTA-3′ R: 5′-ATAGTCTTCTGCGTCCAGGGC-3′	This Work
MDA-5	F: 5′-AGAGCCCGTCCAAAGTGAAGT-3′ R: 5′-AACATCTTCCCCAGAGCAGACT-3′	[30]
TLR3	F: 5′-ACTCGGTGGTGCTGGTCTTC-3′ R: 5′-GAGGAGGCAATTTGGACGAA-3′	[31]
TLR9	F: 5′-TGGGCGTTTGCCAATCTGA-3′ R: 5′-TGTTGAAGCAGGGGAAGCAG-3′	[32]

## Supplementary Materials

The following are available online at https://www.mdpi.com/2076-2607/8/9/1320/s1, Figure S1: Rarefaction curve, Table S1: Microbiota composition, Table S2: Statistical analysis of OTUs abundance.
