# Supplementary material for: Microbiota Modulates the Immunomodulatory Effects of Filifolinone on Atlantic Salmon
Source: Microorganisms. 2020 Aug 30;8(9):1320. doi: 10.3390/microorganisms8091320 (PMC7564783; doi:10.3390/microorganisms8091320)
Supplement: Supplementary file 1 [file microorganisms-08-01320-s001.zip › Figure S1.pptx]

## Slide 1
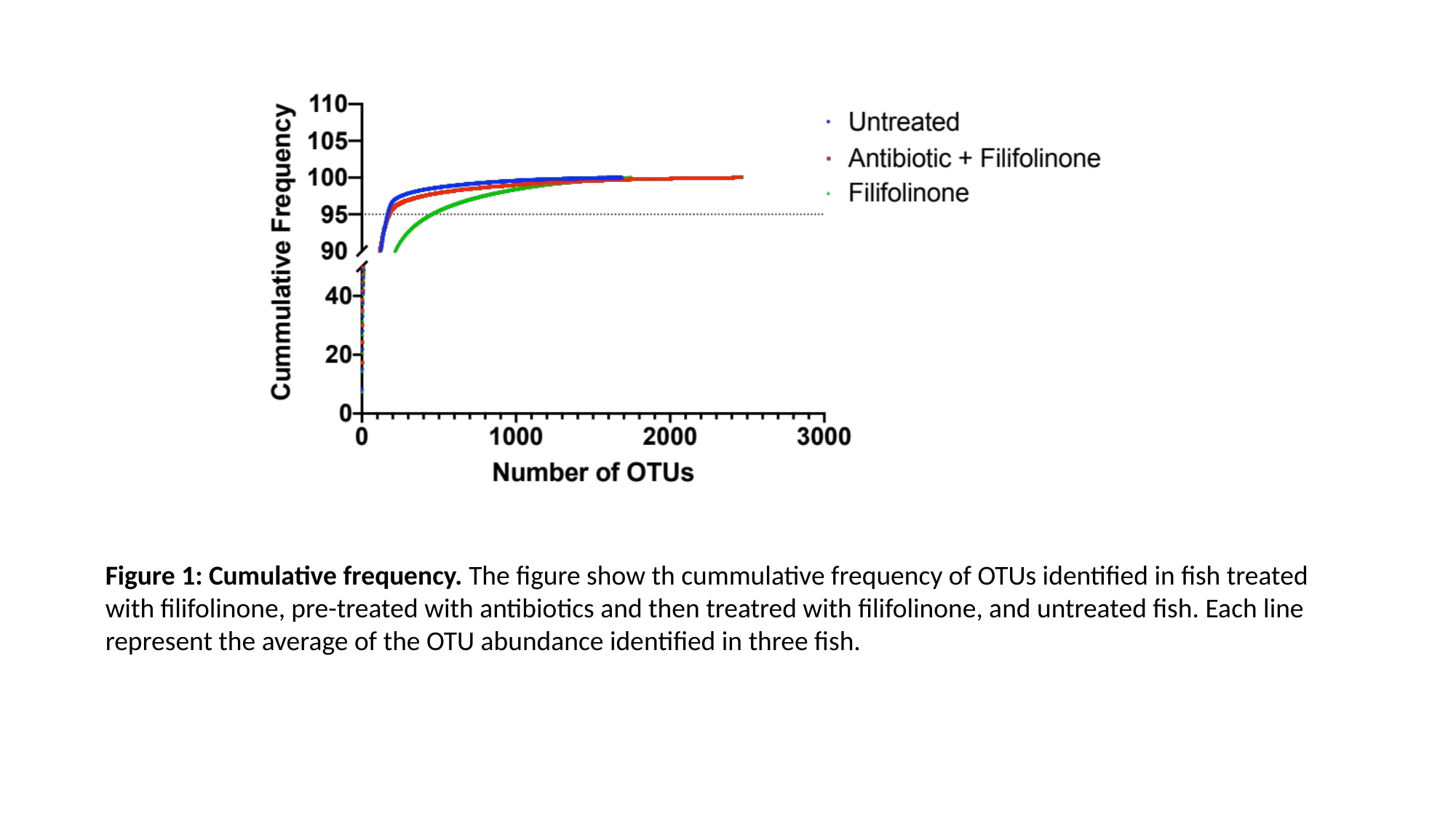

Figure 1: Cumulative frequency. The figure show th cummulative frequency of OTUs identified in fish treated with filifolinone, pre-treated with antibiotics and then treatred with filifolinone, and untreated fish. Each line represent the average of the OTU abundance identified in three fish.
